# Supplementary material for: Highly efficient exciton-exciton annihilation in single conjugated polymer chains
Source: Nat Commun. 2026 Jan 6;17:731. doi: 10.1038/s41467-025-67422-z (PMC12820388; doi:10.1038/s41467-025-67422-z)
Supplement: Supplementary file 1 — Supplementary Information [file 41467_2025_67422_MOESM1_ESM.docx]

**Highly efficient exciton-exciton annihilation in single conjugated polymer chains**

Nicola J. Fairbairn, Olga Vodianova, Bernhard V. K. J. Schmidt and Gordon J. Hedley

School of Chemistry, University of Glasgow, Glasgow, G12 8QQ, United Kingdom

*Email: [Gordon.Hedley@glasgow.ac.uk](mailto:Gordon.Hedley@glasgow.ac.uk) Telephone: +44 (0)141 330 1763

Supplementary Information

**Supplementary Note 1: Experimental Methods**

Single molecule measurements were made on a homebuilt single molecule microscope. Excitation is provided by the 80 MHz output at 800 nm from an ultrafast optical parametric oscillator (Coherent Discovery), this is routed through a pulse picker (APE PulseSelect) to give 40 MHz, with pulses of ~120 fs duration before being frequency doubled with a BBO crystal to give 400 nm. The beam is spatially expanded with a lens pair then converted from linear to circular polarisation with a Berek Compensator (New Focus) set as a quarter waveplate for 400 nm. The beam is coupled into the microscope (Nikon Eclipse Ti2-U), reflected up to the sample with a dichroic (Thorlabs, DMLP425) before being focussed with an objective (Nikon Plan Apochromat λ, 1.45 N.A.). Incident power on the sample is controlled with a neutral density wheel before the beam is expanded, with incident powers ~3 nW at the sample.

The same objective collects emission from single polymer chains. The sample is scanned with a 2D piezo stage (Physik Instrumente, P-733.2CD), with collected emission routed back through the dichroic, through a tube lens onto a pinhole (75 µm) to remove stray light. After recollimation, emission is then directed to the detection setup as described in the main text, with SPADs (MicroPhoton Devices, PD-100-CTE) in either a conventional Hanbury Brown and Twiss geometry with a 50:50 beamsplitter (Thorlabs BS013), or with an additional split between a 458 nm dichroic (Semrock, FF458-Di02) on one of the arms.

Detected photons on the SPADs are recorded with a HydraHarp 400 (PicoQuant) on separate independent channels. Overall measurement control of the piezo and photon counting is made with homecoded software (C, python and Qt framework). The data is stored as raw binary files in a first in, first out (fifo) format, containing full time tags (time with respect to last laser pulse and time with respect to start of measurement) for each photon. Fifo files are analysed in homecoded software (C, python and Qt framework), enabling extraction/analysis of intensities, decays and photon statistics.

**Supplementary Note 2: Sample Preparation for Single Molecule Measurements**

Samples were measured on ~170 µm thick borosilicate glass slides which were cleaned with a mixture of ultrapure water (Merck Direct-Q3) and Hellmanex III to give 2% Hellmanex solution by flushing 3 times followed by 20-minute sonication at 40°C. These steps were repeated another 2 times using ultrapure water. N_2_ was used to dry the slides prior to exposing them to UV ozone twice for 20-minute cycles in a UV ozone cleaner (Novascan, PSD-UV8).

Single molecule PL transients were recorded from individual polymer chains of poly(9,9’-dioctylfluorene) suspended in inert thin film matrices of poly(methyl methacrylate) (PMMA) (approximately 1 x 10^-8^ mg/ml). A 6% by weight 120 kDa PMMA:PFO:toluene mixture was deposited by spincoating in an N_2_ glovebox, giving 200 nm thick films. On the microscope, single chains were measured for 30 seconds each whilst being purged with a constant flow of nitrogen to prevent degradation.

**Supplementary Note 3: Intensity Spot Trace**

The location of each polymer chain was determined by recording images generated by scanning the piezo over 20x20 µm regions, as shown in Supplementary Figure 1.


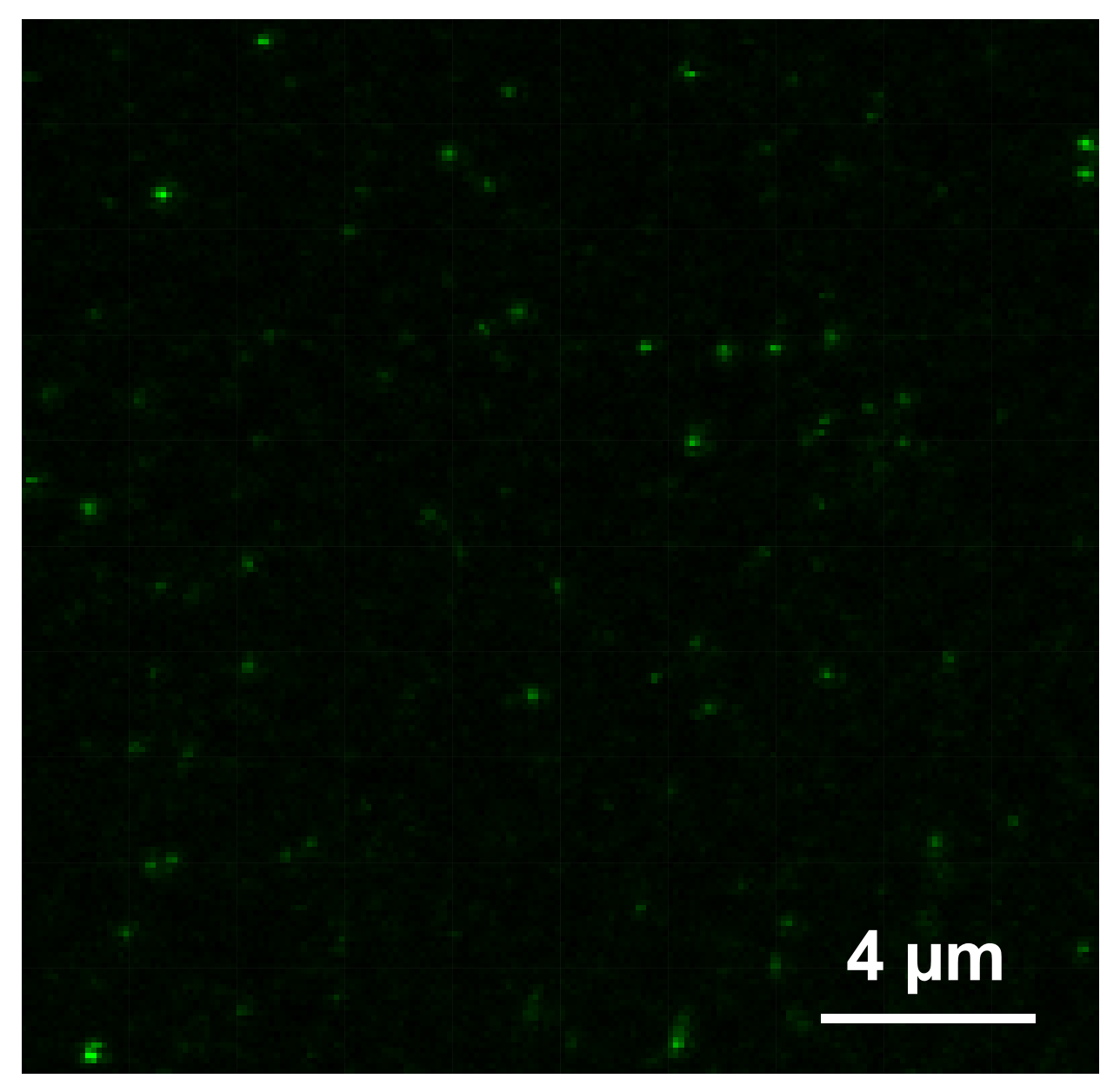


**Supplementary Figure 1:** PL scan image of a 20x20 µm region of a single molecule concentration PFO sample doped in PMMA. The bright green spots are individual PFO chains.

Each polymer chain can be identified as a single diffraction-limited spot. Once all spots within an image were located, they could be measured by moving the laser to each spot for a full 30 seconds, producing PL transients as a function of time, as shown in Supplementary Figure 2 below.


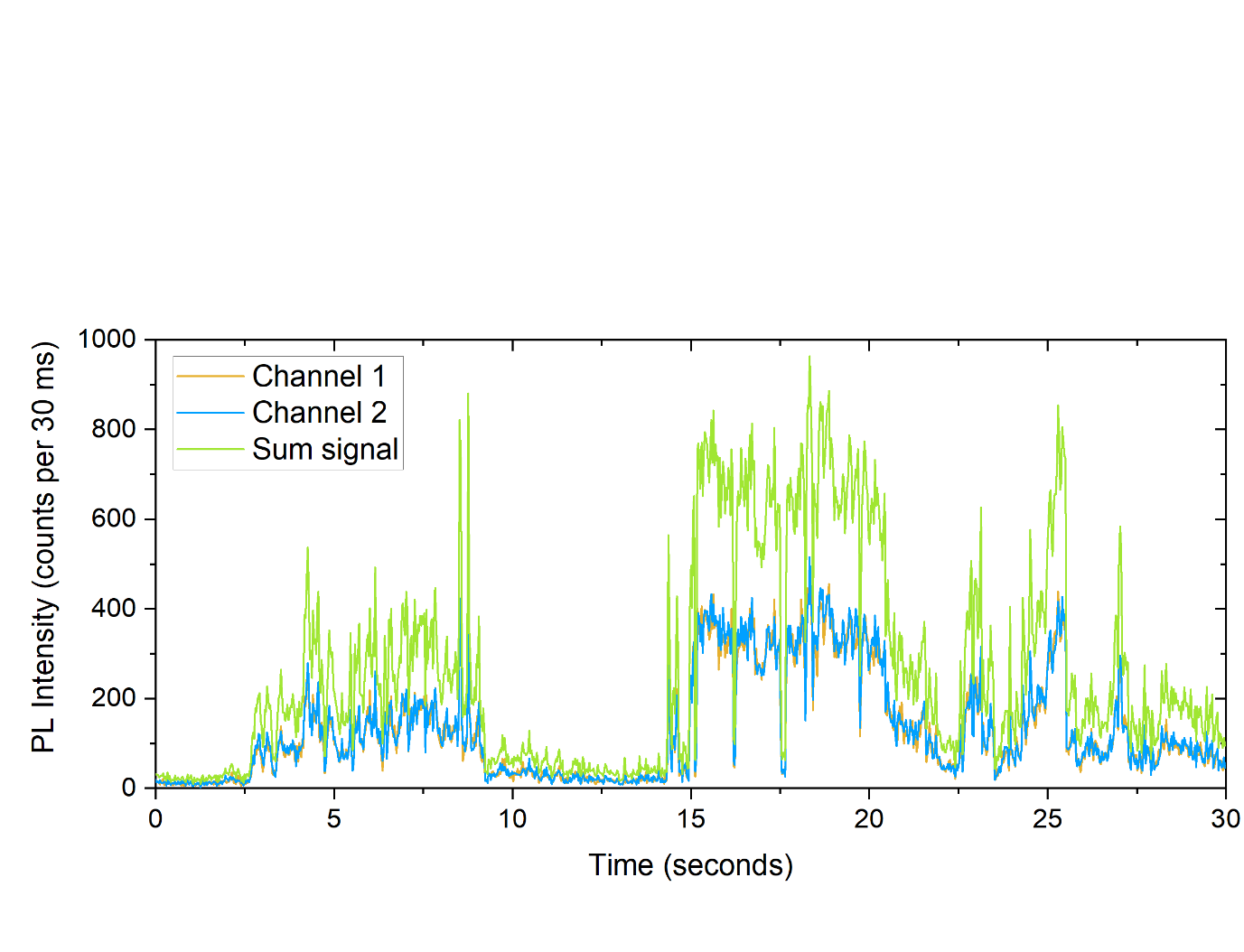


**Supplementary Figure 2:** PL intensity traces of a single PFO chain measured over 30 seconds with a binning of 30 ms. Two detectors in a Hanbury Brown and Twiss geometry are used here, with each individual and the sum of them shown.

**Supplementary Note 4: Simulation of Expected Colour Ratio from PL Spectra**

Optical simulations were performed by taking an emission spectrum consisting of an array of wavelengths and performing spectral multiplication with all the optical components that modify this emission before it is recorded on both detectors. These components are: the microscope objective transmission, the 425 nm dichroic filter in the microscope, the non-polarising 50:50 beamsplitter, the 458 nm dichroic mirror and the photon detection efficiency of the detector, Supplementary Figure 3. This information was obtained from specification sheets of the specific components, with part numbers as listed above in the experimental methods. Both channels’ detection was distinguished by reflectance or transmittance through the 458 nm dichroic mirror.


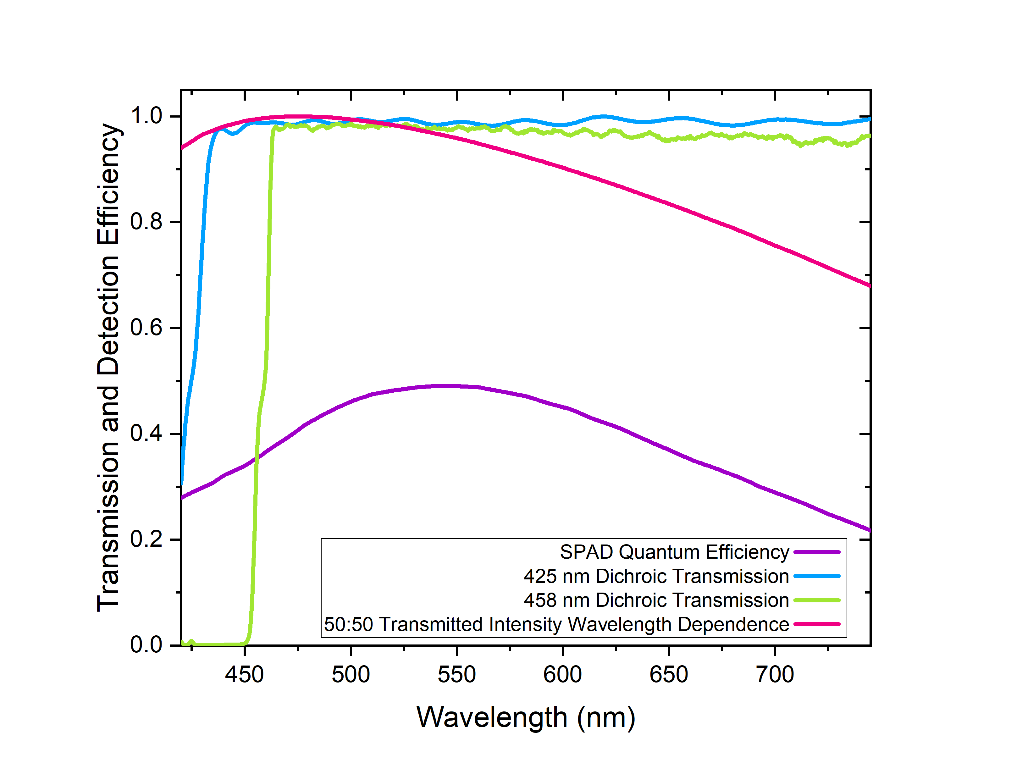


**Supplementary Figure 3:** Relative efficiency of each respective optical component used for both the detection of the colour ratio and optical simulation of the emission spectra.

Multiplying these components together gives channel intensity values detected on transmission through the 458 nm dichroic mirror onto the first detector. To calculate the channel intensity values detected on the second detector by reflectance, the 458 nm dichroic mirror component spectrum is subtracted from 1, and this ‘reflectance’ spectrum is multiplied together with the remaining optical component spectra.

Spectral multiplication of these curves therefore results in the final wavelength-dependence sensitivity on each detector, where the dichroic transmission corresponds to redder wavelengths (λ > 458 nm) whilst the reflectance (otherwise regarded as 1 – transmission) corresponds to bluer wavelengths (λ < 458 nm), as shown in Supplementary Figure 4.


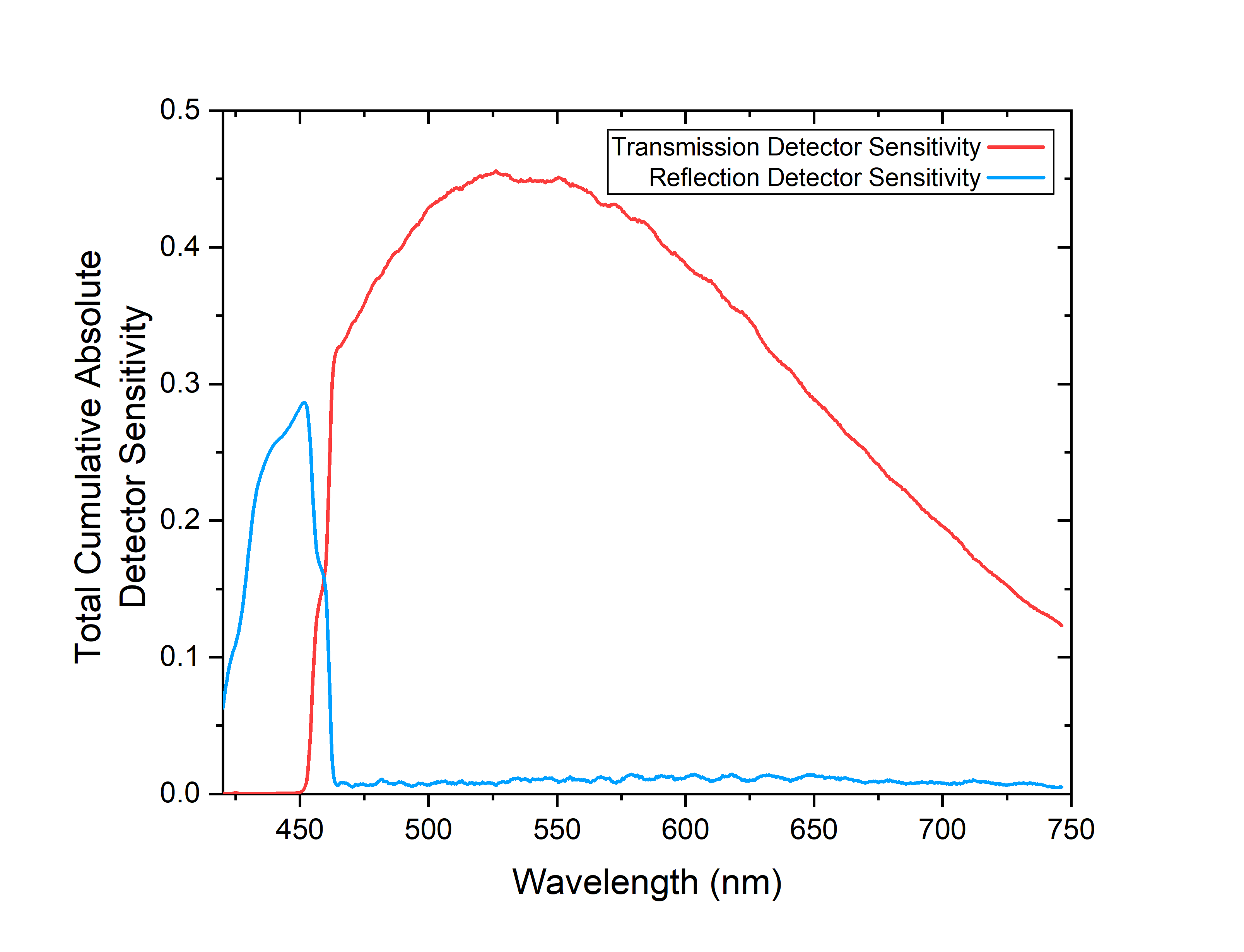


**Supplementary Figure 4:** Total cumulative absolute detector sensitivity for the two detectors (one in the transmission position, one in the reflection) when incoming PL is split with the 458 nm dichroic filter.

By taking a measured PL spectrum and multiplying it by the two final SPAD sensitivity plots from Supplementary Figure 4 above, we can observe the overall intensity values that would be recorded on each detector, one in transmission geometry, one in reflection. For conventional PFO emission, corresponding to steady state, this gives the two spectra shown in Supplementary Figure 5 below for each detector. Integrating the two curves gives two intensity values ($I_{\lambda>458}$ and $I_{\lambda<458}$) that can be used to calculate the colour ratio, where,

$$Colour Ratio= \frac{I_{\lambda>458}-I_{\lambda<458}}{I_{\lambda>458}+I_{\lambda<458}}$$

For steady state PFO this gives a colour ratio of -0.24.


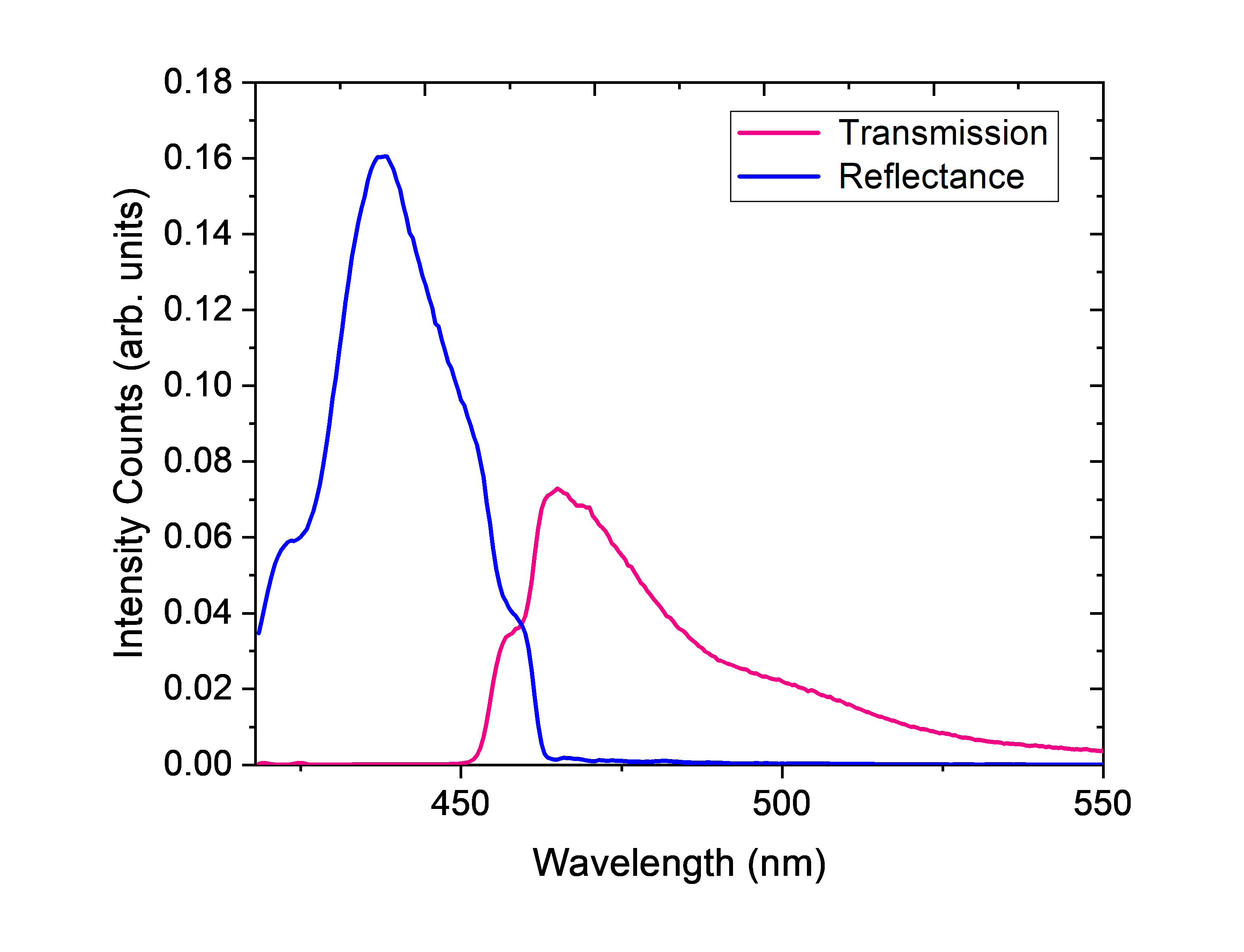


**Supplementary Figure 5:** Simulated overall intensity values recorded on each SPAD for steady state PFO emission. The two are integrated to give a calculated colour ratio of -0.24.

For beta-phase PFO, the simulated detected spectra were calculated, shown in Supplementary Figure 6, giving a colour ratio of 0.008.


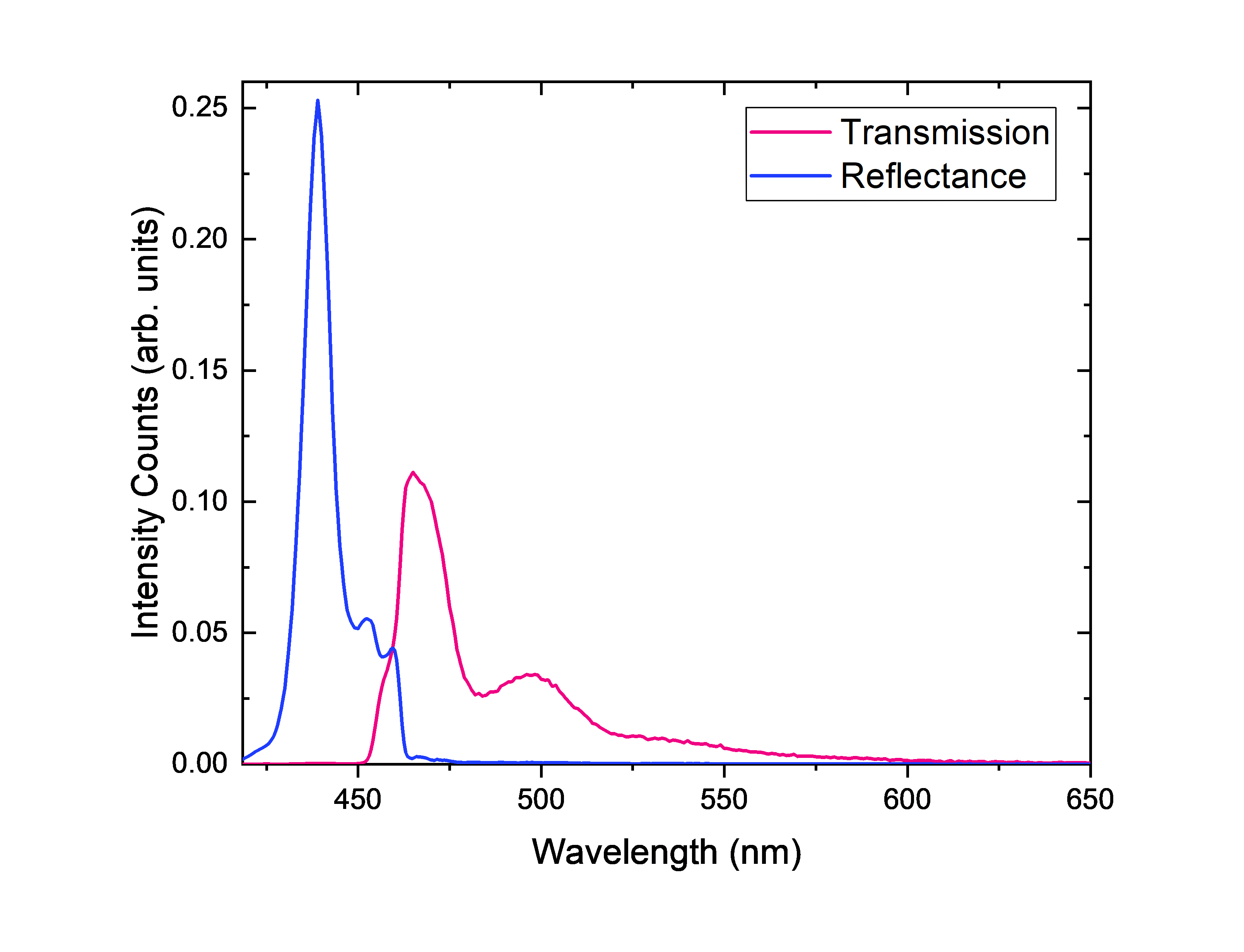

**Supplementary Figure 6:** Optically simulated overall intensity values of beta-phase PFO emission recorded on each SPAD. The two are integrated to give a calculated colour ratio of 0.008.

While for fluorenone keto-defects, a PL spectrum of solely it (from reference 48 of the main text) gives the two simulated spectra shown in Supplementary Figure 7 below. Here the colour ratio is calculated to be 0.945.


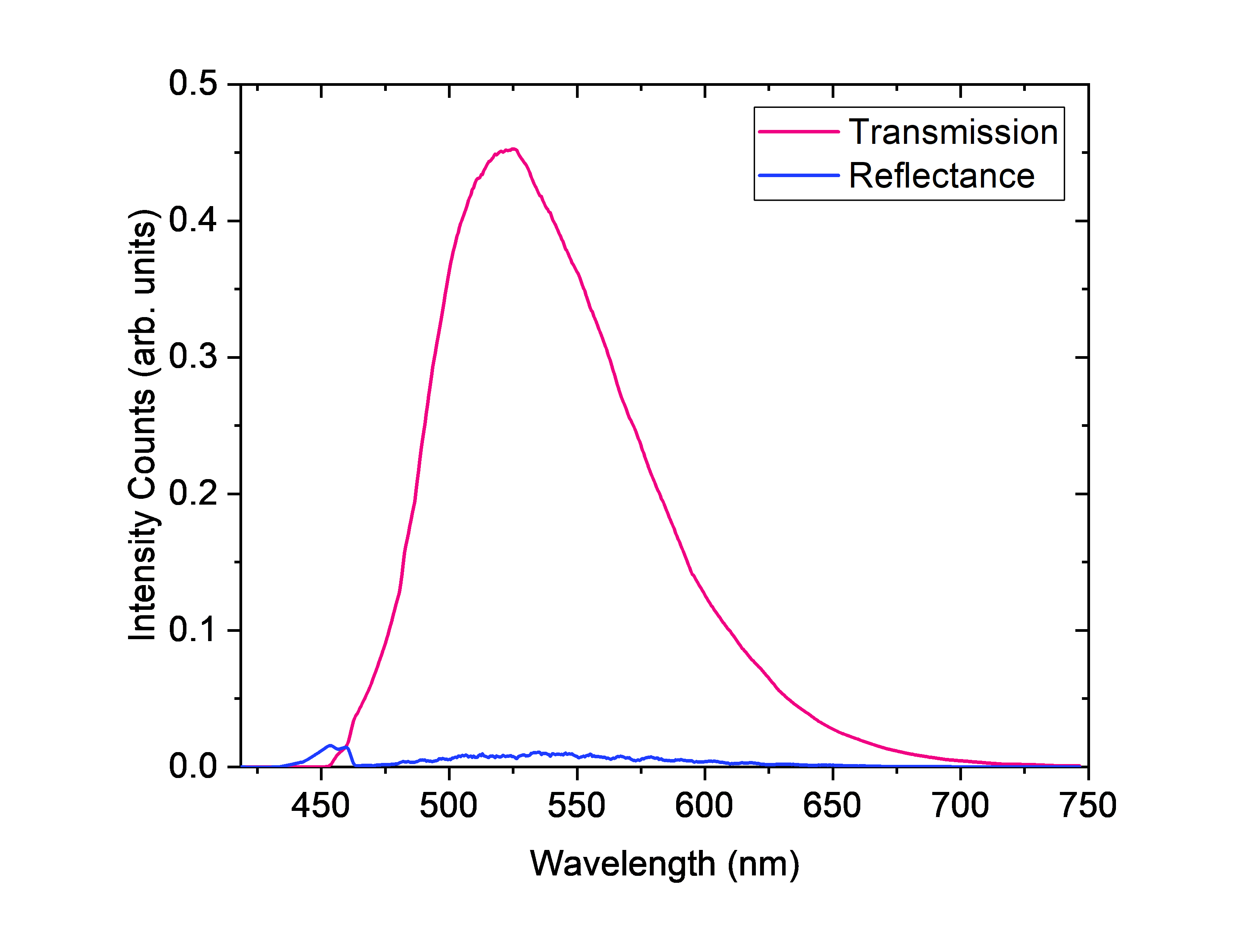


**Supplementary Figure 7:** Optically simulated overall intensity values of fluorenone emission recorded on each SPAD. The two are integrated to give a calculated colour ratio of 0.945.

**Supplementary Note 5: Colour Ratio Binning**

Figure 3c of the main text shows measured values of the colour ratio across 5012 chains of PFO. This data is extracted with an intensity condition that at least 200 counts are present in the sum between the two colour ratio detectors (detectors 1 and 2 in Figure 3) in each 20 ms bin. All 20 ms slices that have less than 200 counts are discarded. This method is implemented in the analysis software and is done to ensure that any 20 ms periods of time when the polymer chain is emitting little or no light are not recorded in the colour ratio histogram. Additionally, this condition ensures that a high resolution of the colour ratio histogram can be shown (with a binning of 0.01), as any 20 ms slices with fewer counts would cluster around specific values, e.g. a 20 ms slice with 20 counts could only have 21 values of: -1, -0.9, -0.8 … 0.8, 0.9, 1 and thus lead to apparent spikes in the summated distribution across all chains. This ≥ 200 count condition is also used for all subsequent antibunching/TRAB calculations, namely only 20 ms time slices with at least 200 counts are used for the results plotted in Figure 3d, as discussed below.

**Supplementary Note 6: Spectrally-Resolved Time-Resolved Photon Antibunching**

To calculate the time-resolved photon antibunching (Figure 3d) we have developed software routines to process the raw photon stream, extracting information of interest. The ≥ 200 count condition described above is first used to identify 20 ms time slices, and the colour ratio for each of them is calculated. TRAB is then performed on the photons in this time slice, with the photons in channels 1 and 2 (the colour ratio arm) treated as if they come from a single new virtual channel which is correlated with those from detector 3 (the other 50% of detected photons). In effect, the colour ratio arm allows us to ascertain the colour ratio for valid 20 ms slices and assign the resultant photon statistics to different spectral regions. An obvious trade-off exists when spectrally resolving photon antibunching, as the higher the spectral resolution the fewer photons will be in any correlation. Here we chose three large regions for spectral sampling, with colour ratio windows of -0.6 to 0.1, 0.2-0.7 and 0.8-1 being used.

**Supplementary Note 7: Comparison between 2 detector non-colour ratio and 3 detector colour ratio TRAB**


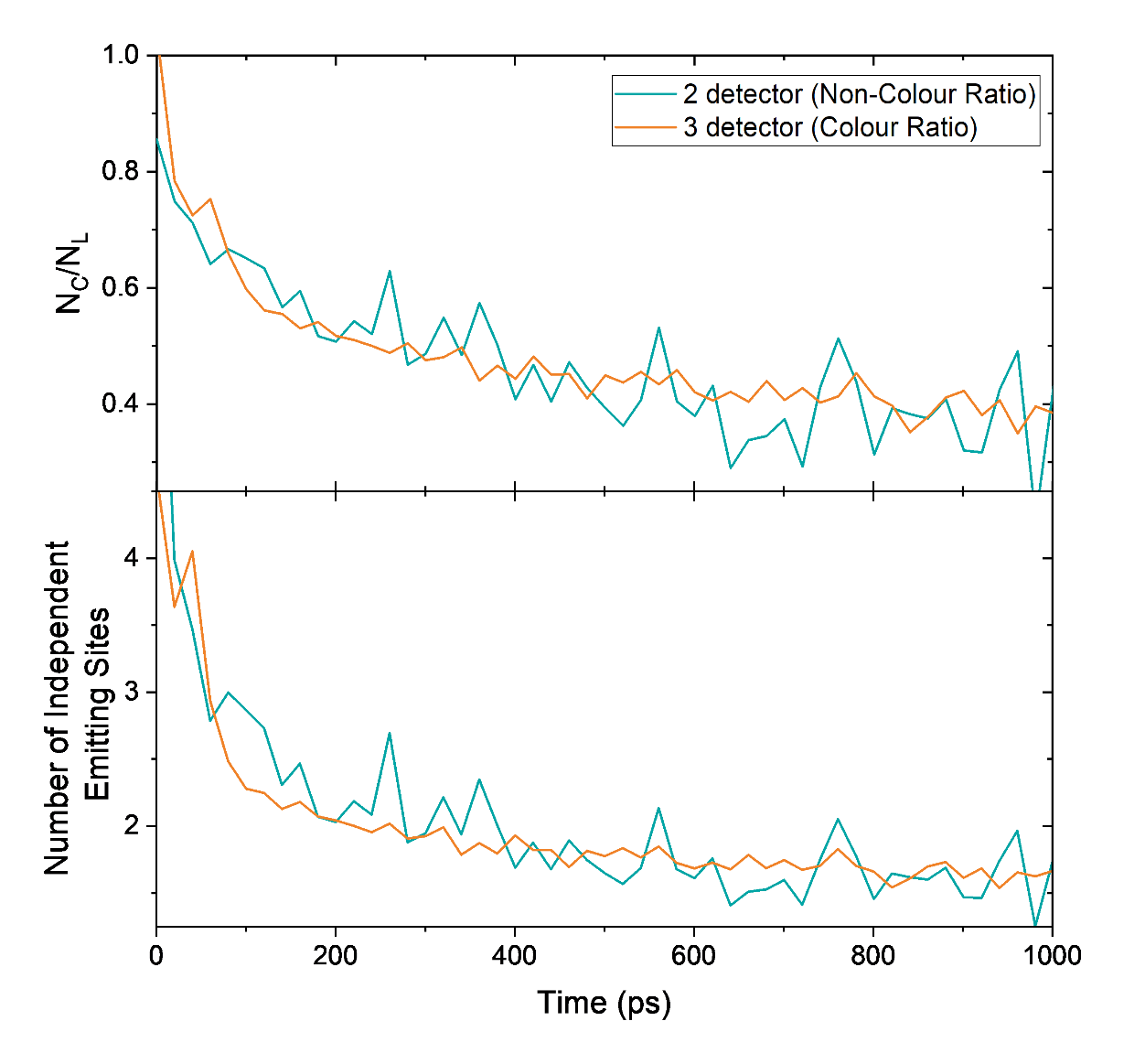


**Supplementary Figure 8:** Direct comparison between the 2 detector (non-colour ratio) TRAB measurements as shown in Figure 2 of approximately 2215 chains and 3 detector (colour ratio) TRAB measurement of 5012 chains. Only the spectrally filtered TRAB is shown in Figure 3 of the main text.

The direct comparison of TRAB measurements for both the non-colour ratio group of 2215 chains and the colour ratio group of 5012 chains (without any spectral filtering) is shown in Supplementary Figure 8. This is plotted as both *N*_C_/*N*_L_ and as number of emitters as a function of time to enable direct comparison of the two. It is observed that they follow each other very closely, indicating that we are observing the same behaviour, regardless of the different optical setup in the three-detector measurement.

**Supplementary Note 8: Spectrally- and Brightness- Resolved Time-Resolved Photon Antibunching**

To explore how the brightness of the chains affects the spectral- time-resolved antibunching we first need to identify suitable brightness ranges to study. To do this the measured time traces from all 5012 chains from Figure 3 were plotted with 20 ms binning and the brightest 20 ms slice for each of them was stored. This data was then histogrammed to give us an overall brightness histogram, as shown below in Supplementary Figure 9.


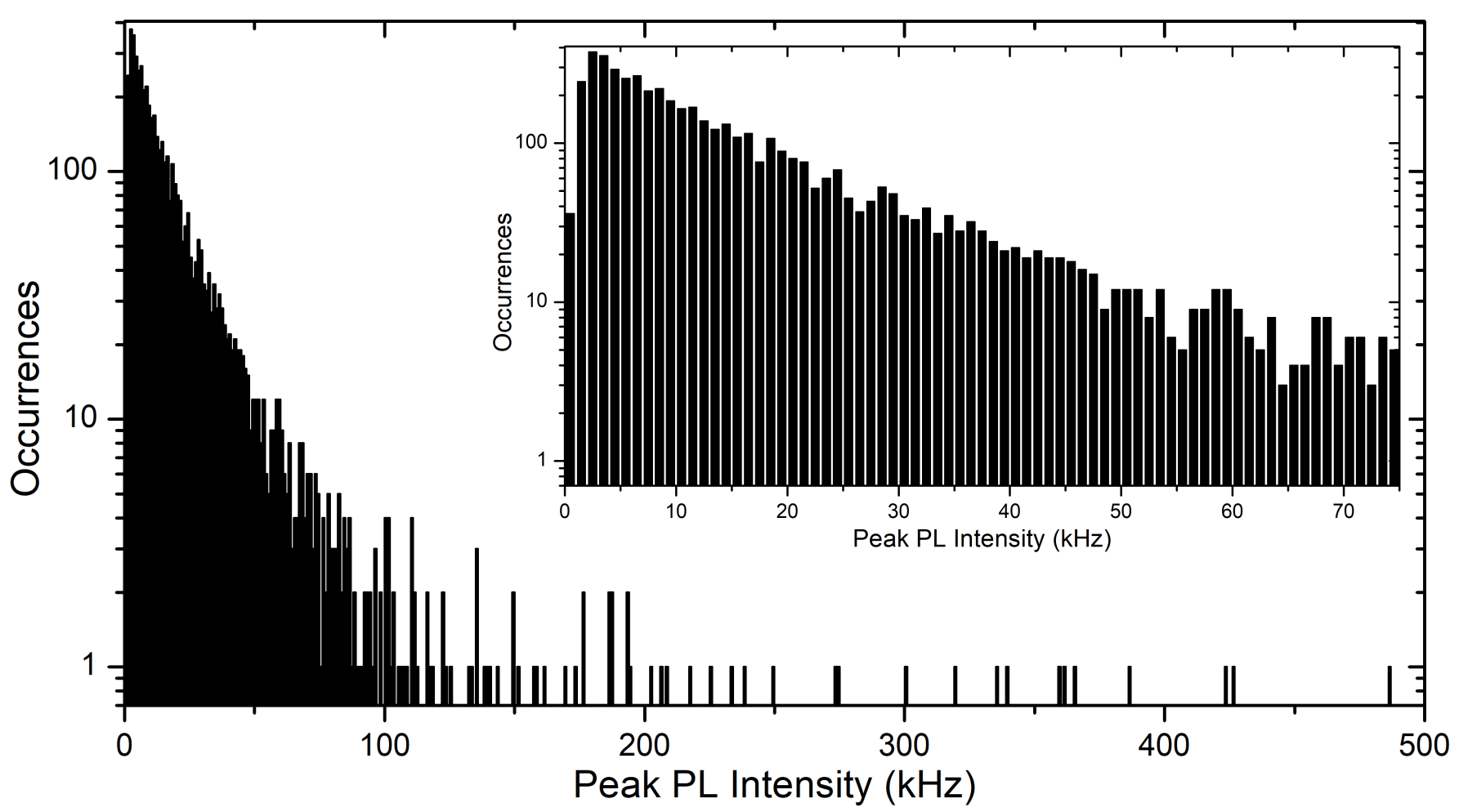


**Supplementary Figure 9:** Histogram of maximum counts per 20 ms found in each chain for all 5012 chains of PFO. Inset is shown zoomed in of the region 0-75 kHz.

Now we find four regions to use as intensity filters for the TRAB calculations (on the three colour ratio regions, blue, yellow and red as per Figure 3). We identified 0-25, 25-50, 50-75 and 75+ kHz as the four regions. The TRAB plots for each of these for the three colour regions are shown below in Supplementary Figures 10-12.


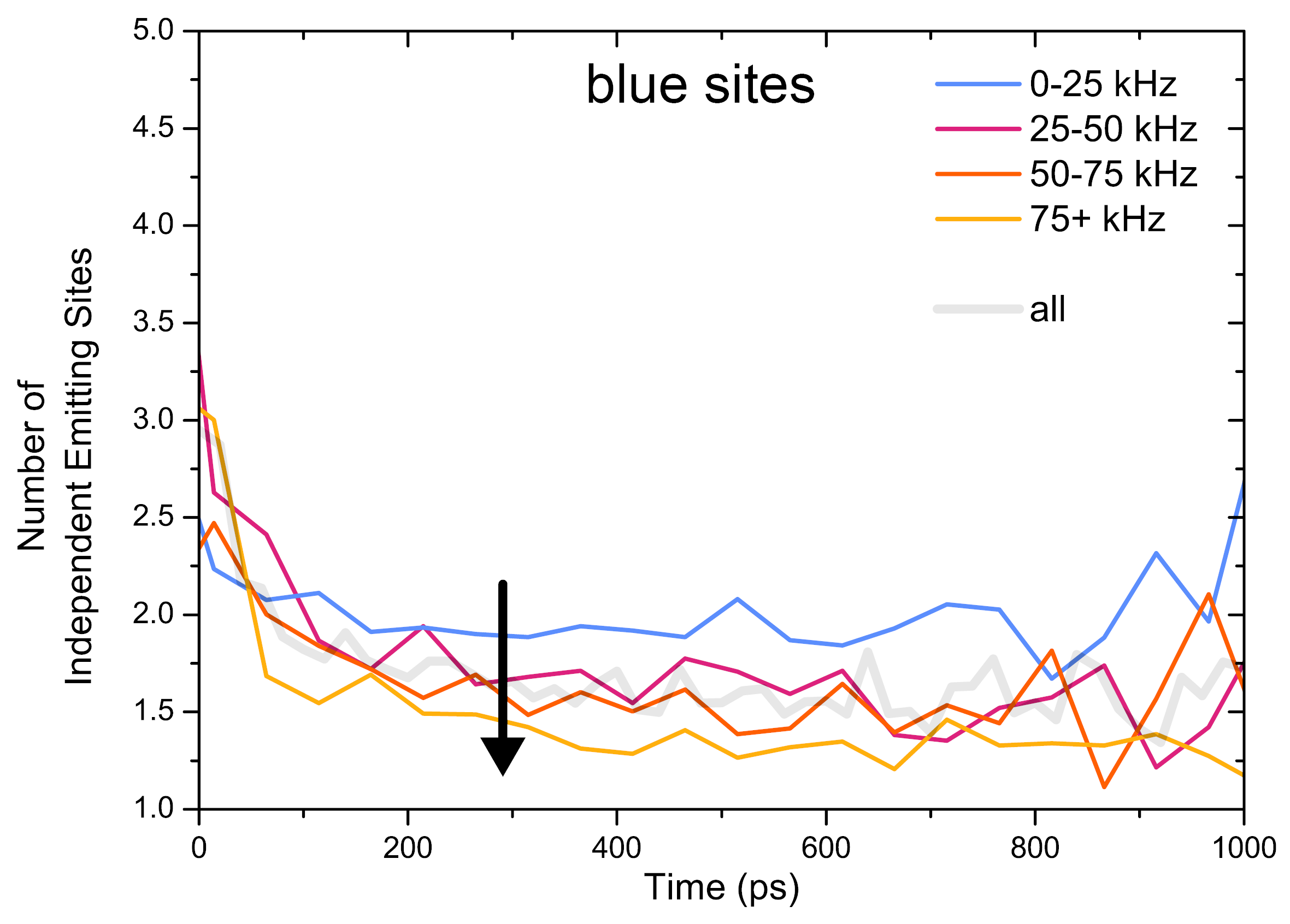


**Supplementary Figure 10:** TRAB plot for four filtered intensity ranges as shown, for blue sites (colour ratio -0.6 to 0.1) with 20 ms slicing.


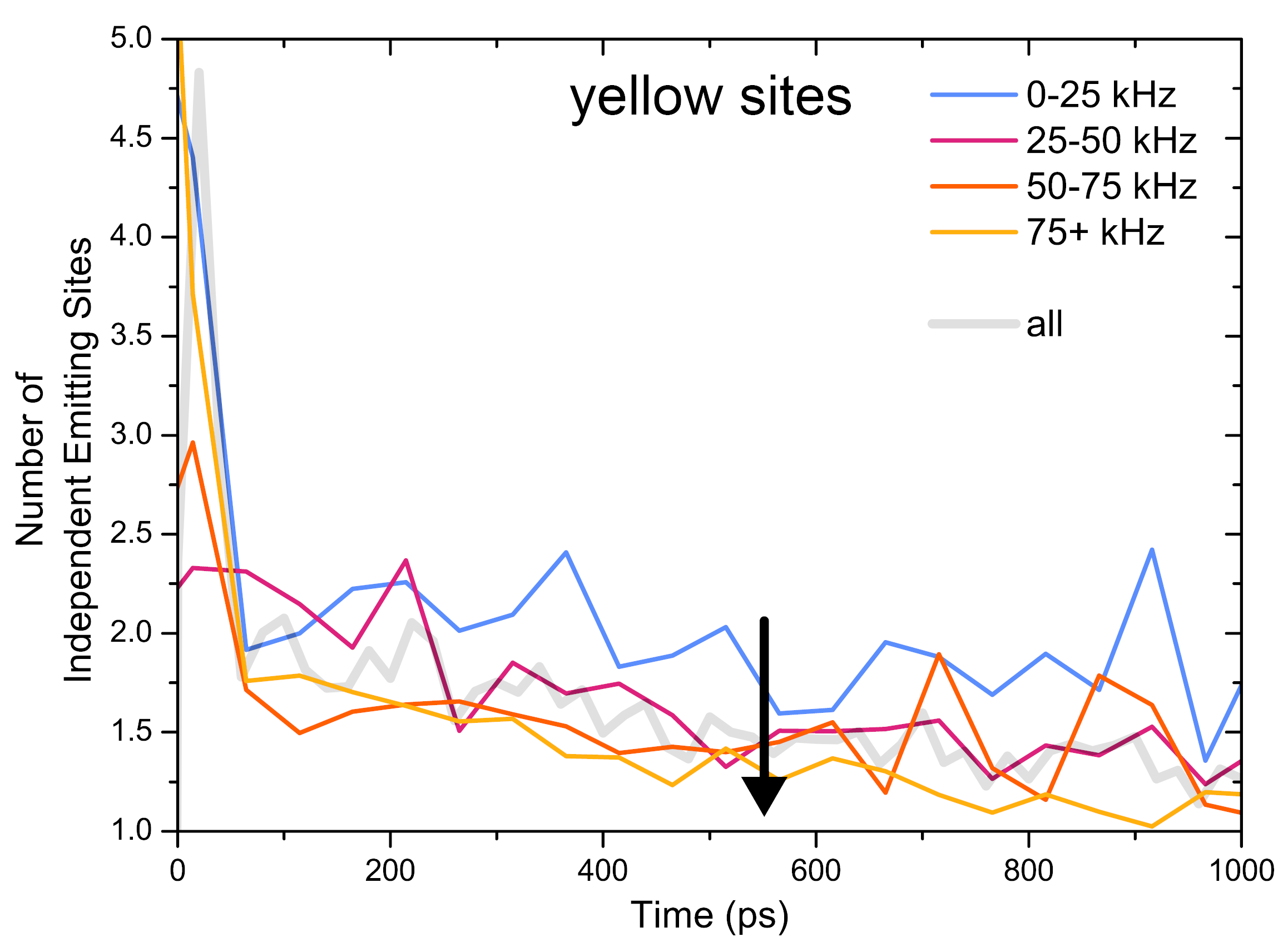


**Supplementary Figure 11:** TRAB plot for four filtered intensity ranges as shown, for yellow sites (colour ratio 0.2 to 0.7) with 20 ms slicing.


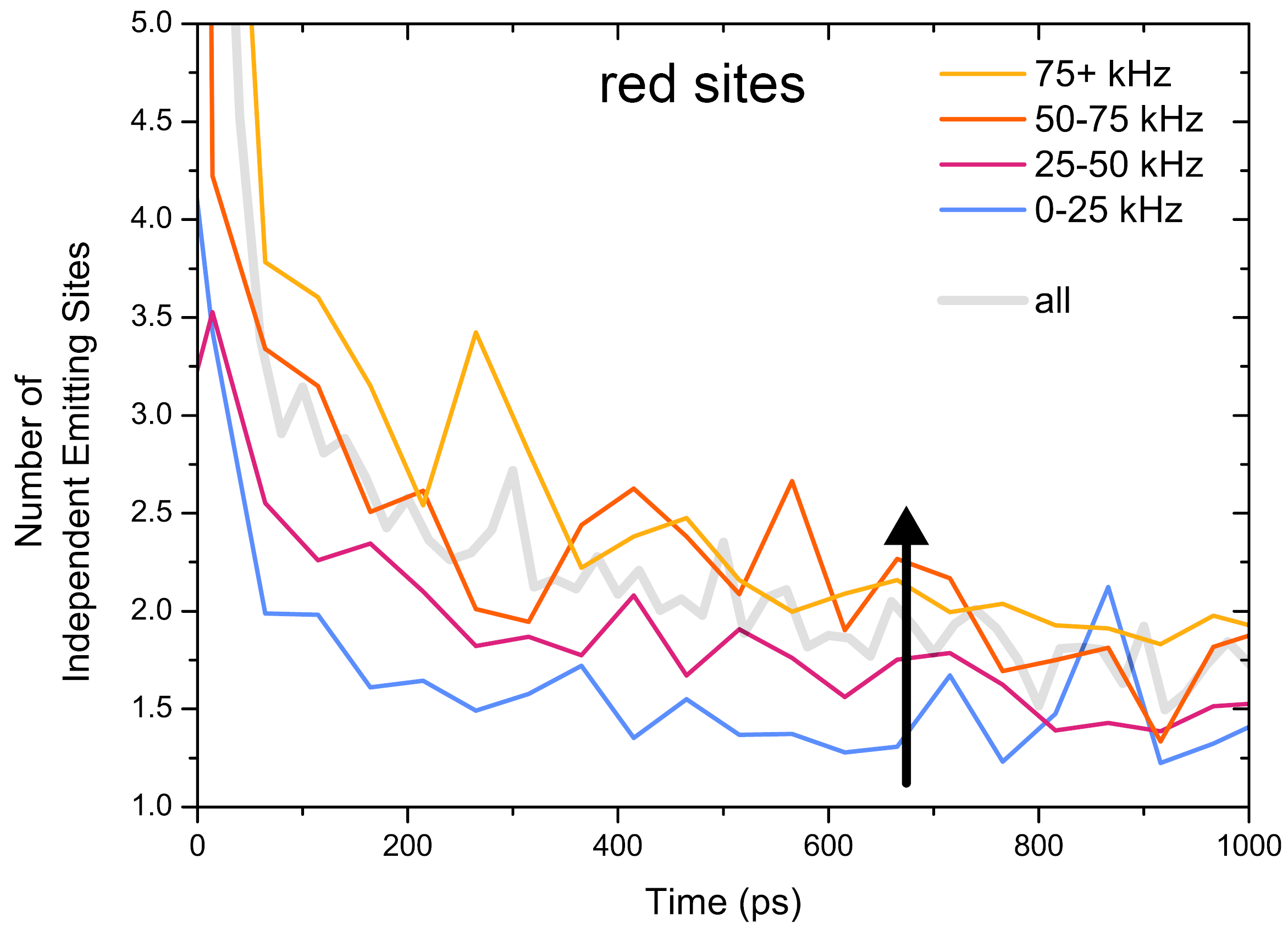


**Supplementary Figure 12:** TRAB plot for four filtered intensity ranges as shown, for red sites (colour ratio 0.8 to 1) with 20 ms slicing.

**Supplementary Note 9: 1-sigma confidence intervals on Spectrally-Resolved Time-Resolved Photon Antibunching**

To confirm that there is a clear and measurable difference between each of the datasets plotted in Figure 3d, we can construct a running error on the *N*_C_/*N*_L_ ratio (which is then converted into an error on the number of independent sites via main text equation 1). The standard deviation is calculated for each *N*_L_ (*σN*_L_) and then applied to maximise the upper and lower error bounds, i.e. (*N*_C_ + *σN*_L_)/ (*N*_L_ - *σN*_L_) for the largest possible number of emitters, and (*N*_C_ - *σN*_L_)/ (*N*_L_ + *σN*_L_) for the smallest number. As we have no way to measure *σN*_C_, we use the *σN*_L_ value as a substitute. We then plot these error bounds as an area above and below the main curves, as shown below in Supplementary Figure 13.


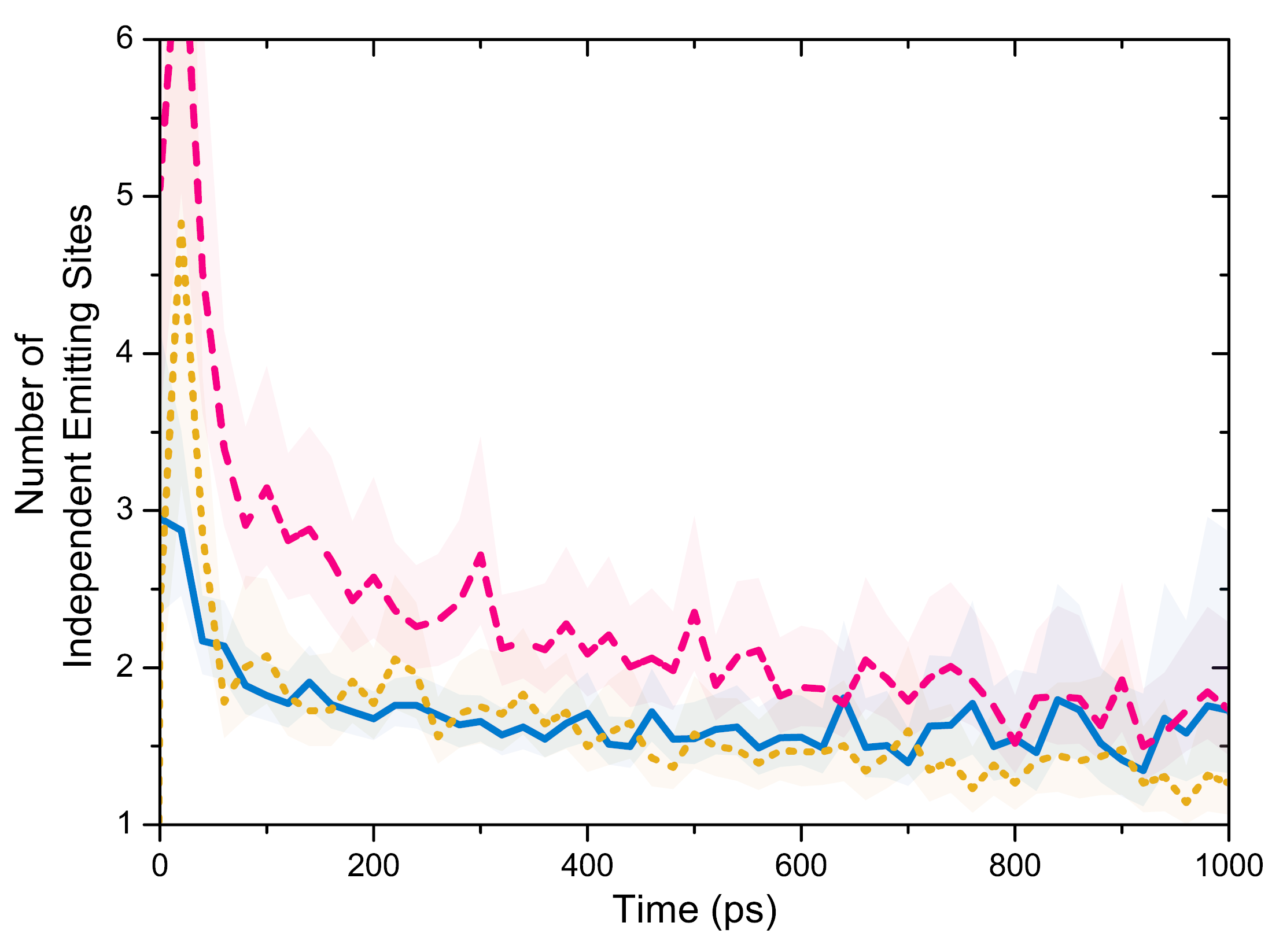


**Supplementary Figure 13:** TRAB plot as shown in Figure 3d, but with error bounds for each colour ratio, showing the 1*σ* confidence interval. The error bound areas have been made semi-transparent to aid identification when they are overlapping.

**Supplementary Note 10: Instrument Response Function of the TCSPC setup**


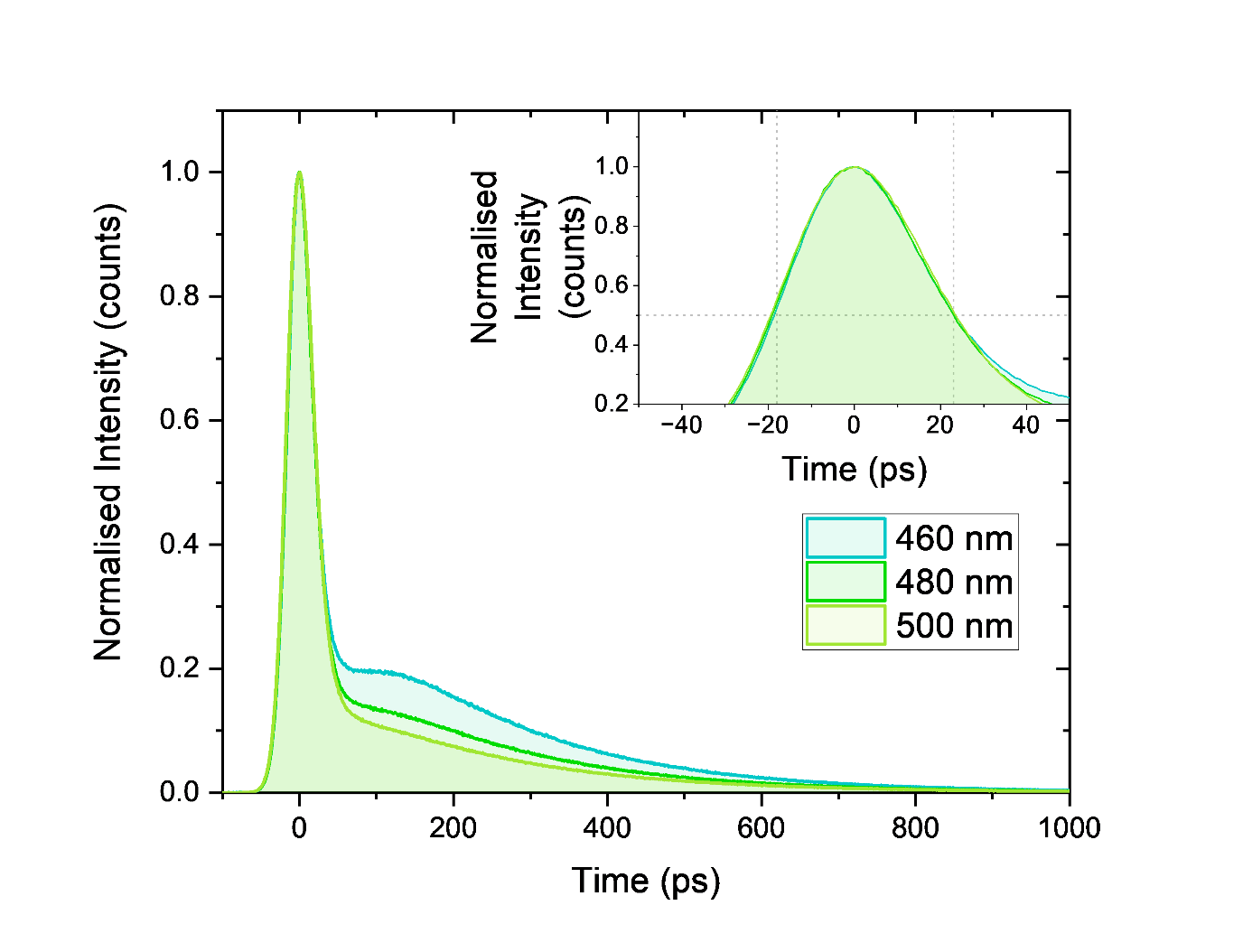


**Supplementary Figure 14:** Normalised instrument response functions of the TCSPC setup when using direct fs laser scatter for 460 nm, 480 nm and 500 nm wavelengths, measured at 40 MHz and with 1 ps binning. Inset is a zoomed-in region around the peak, showing a full-width half-maximum of ~40 ps.

**Supplementary Note 11: Variation of Single Chain Colour Ratio Traces**

For each of the 5012 measured chains colour ratio traces can be calculated. In Figure 3b we show one that covers the full range of values with relatively frequent changes. However, a range of behaviours is present. Shown below in Supplementary Figure 15 are three that show: red and then yellow sites dominating before a switch to blue sites at 6 seconds (top), blue sites with very occasional jumps to yellow and red sites (middle) and fast switching between red and blue for 12 seconds, then red site domination until the end of the trace (bottom).


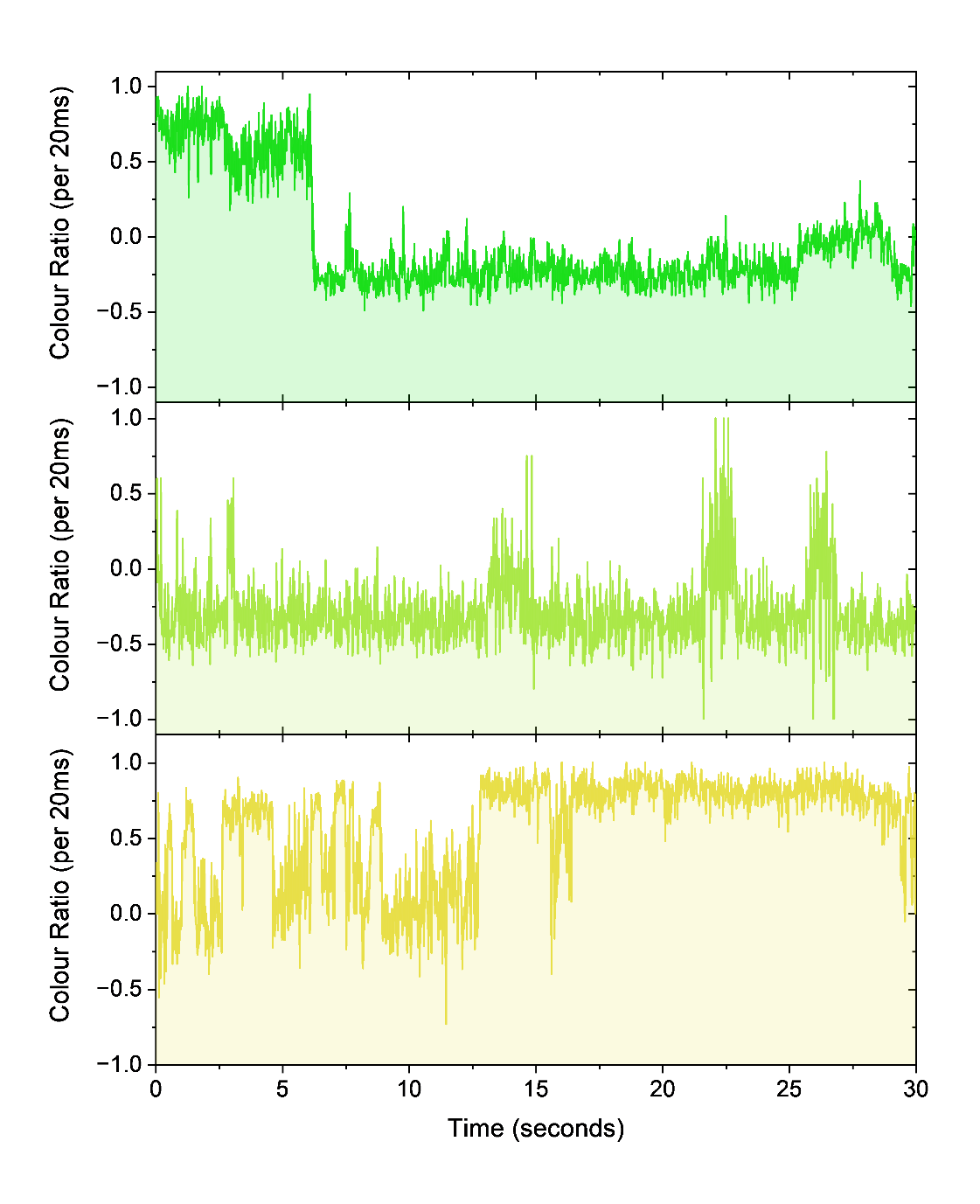


**Supplementary Figure 15:** Colour ratio traces over 30 seconds for three separate single chains binned at 20 ms time resolution.

**Supplementary Note 12: Time-window Dependent and Count Dependent Spectrally-Resolved Time-Resolved Photon Antibunching**

The spectrally-resolved time-resolved antibunching shown in Figure 3d is calculated for individual 20 ms time slices, each with ≥ 200 counts present. To test how sensitive the observed decay in the number of independent emitting sites is for blue, yellow and red sites as a function of this time slice window, or target counts, different traces can be calculated.

Firstly, the time slicing was varied from the original 20 ms to 10, 40 and 200 ms. To ensure the same intensity ranges are being used for the correlation calculations the target counts was scaled accordingly (from the original ≥200 counts to ≥100, ≥400 and ≥2000 respectively). Almost no change is observed, as shown in Supplementary Figures 16-18 below.


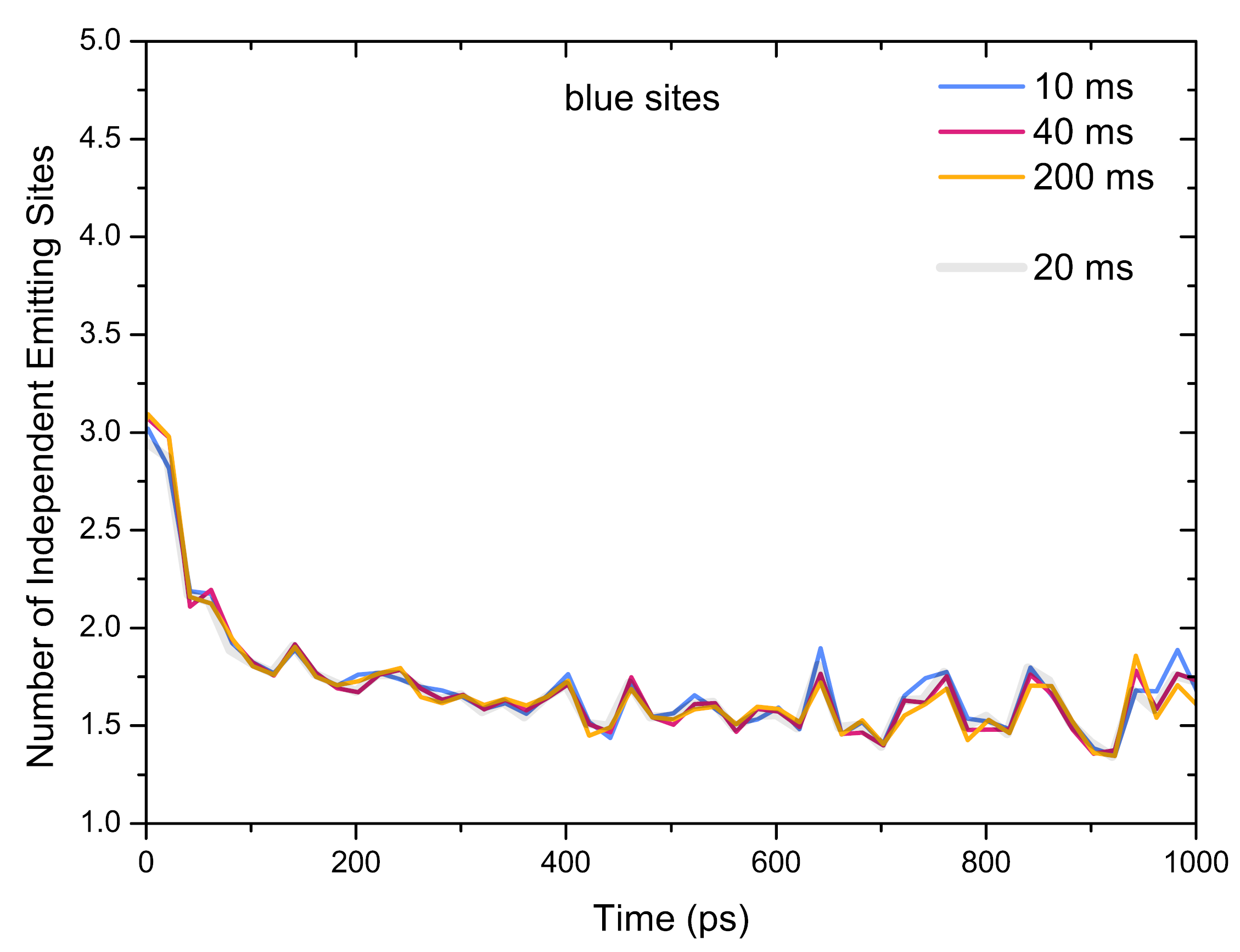


**Supplementary Figure 16:** TRAB plot for blue sites (colour ratio -0.6 to 0.1) for three different time slice windows, and the originally used 20 ms.


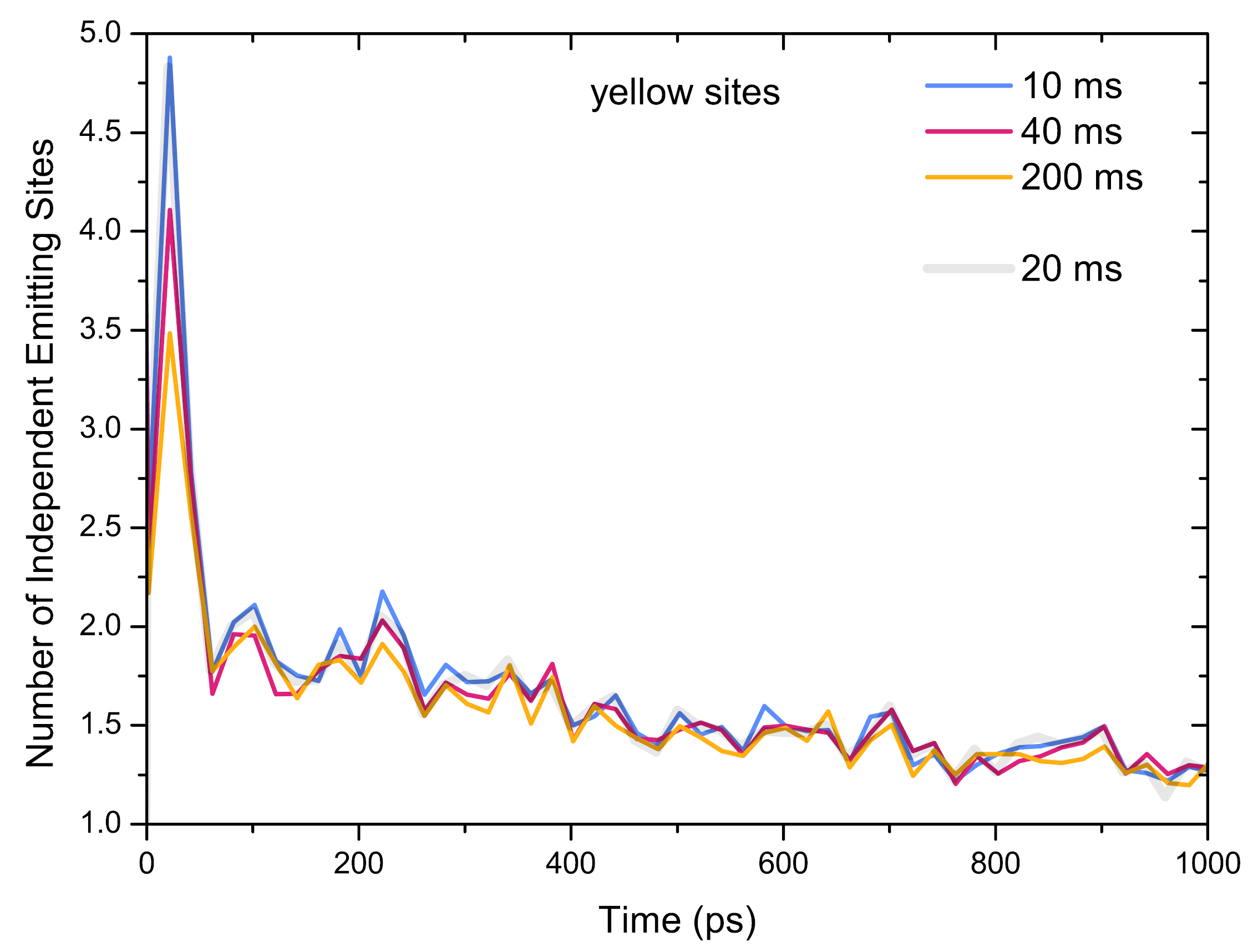


**Supplementary Figure 17:** TRAB plot for yellow sites (colour ratio 0.2 to 0.7) for three different time slice windows, and the originally used 20 ms.


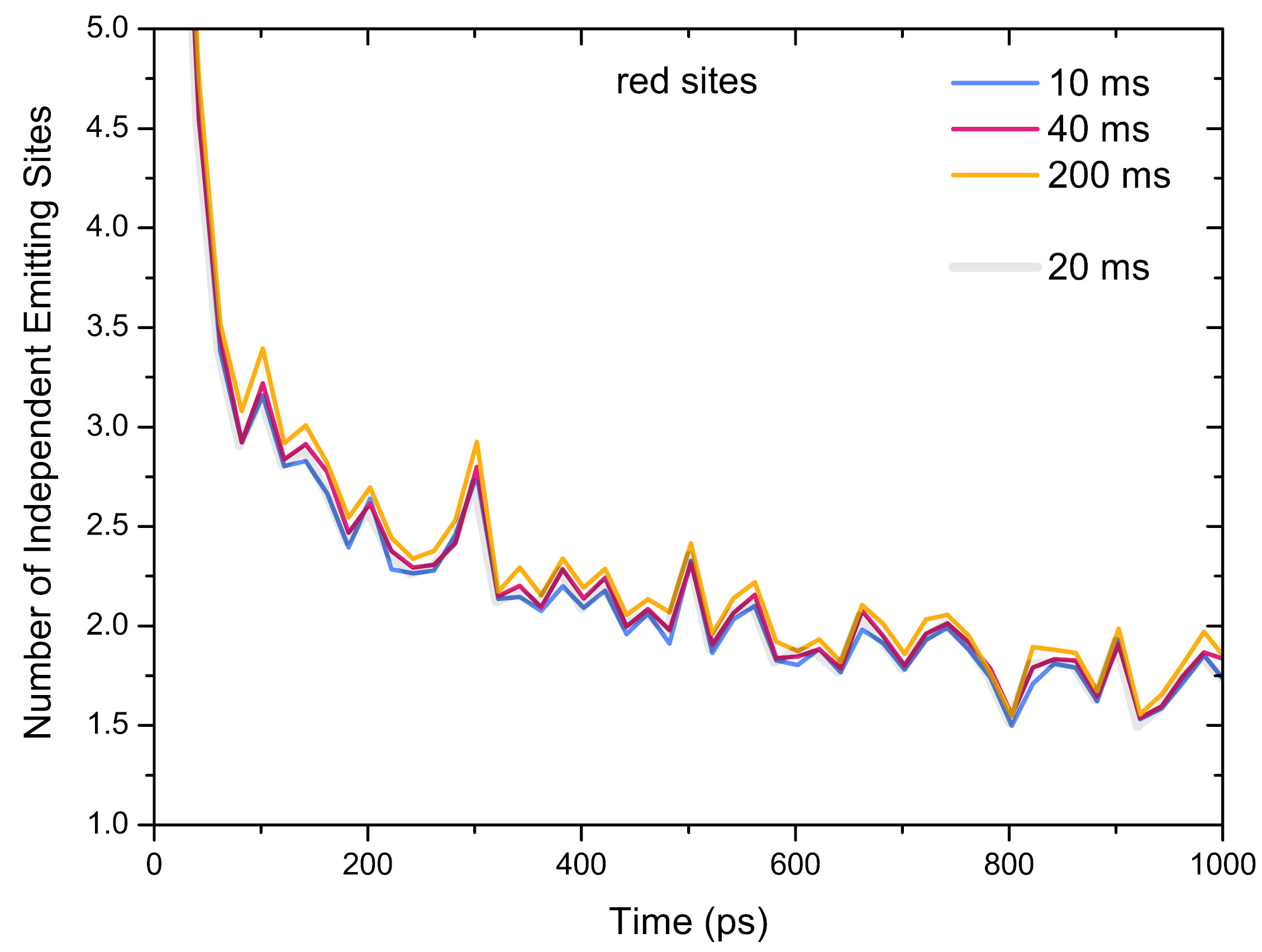


**Supplementary Figure 18:** TRAB plot for red sites (colour ratio 0.8 to 1) for three different time slice windows, and the originally used 20 ms.

Secondly, the count threshold (for 20 ms slices) was removed and compared with the ≥ 200 original, with very little difference observed, as shown in Supplementary Figures 19-21 below. We did not go to higher thresholds as that would essentially be partly replicating the intensity filtering shown in Supplementary Figures 10-12.


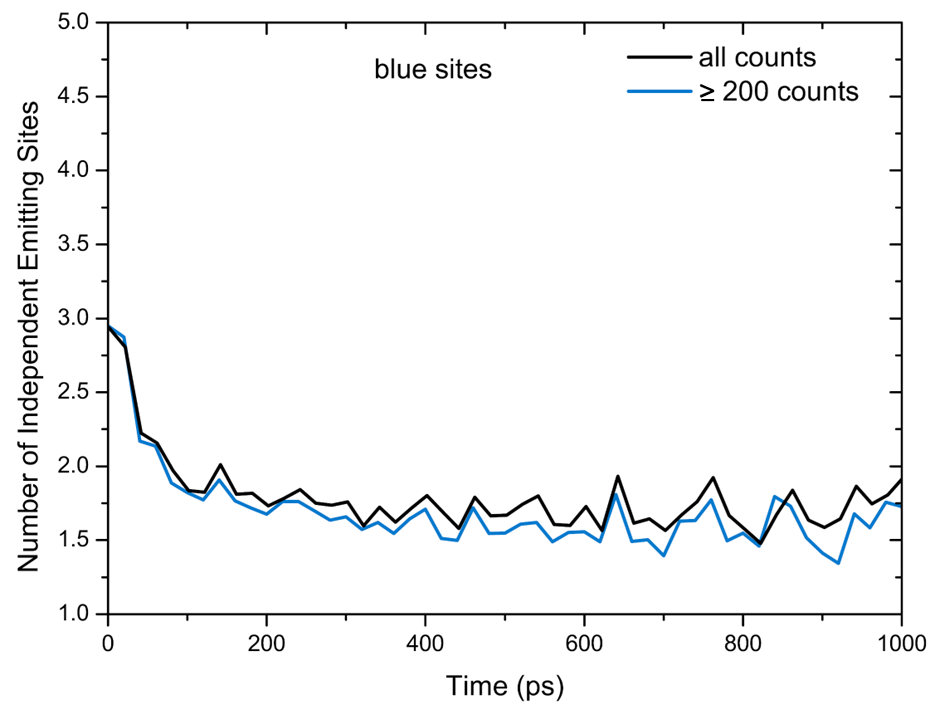


**Supplementary Figure 19:** TRAB plot for blue sites (colour ratio -0.6 to 0.1) for two count conditions (all photons per 20 ms and ≥ 200 photons per 20 ms).


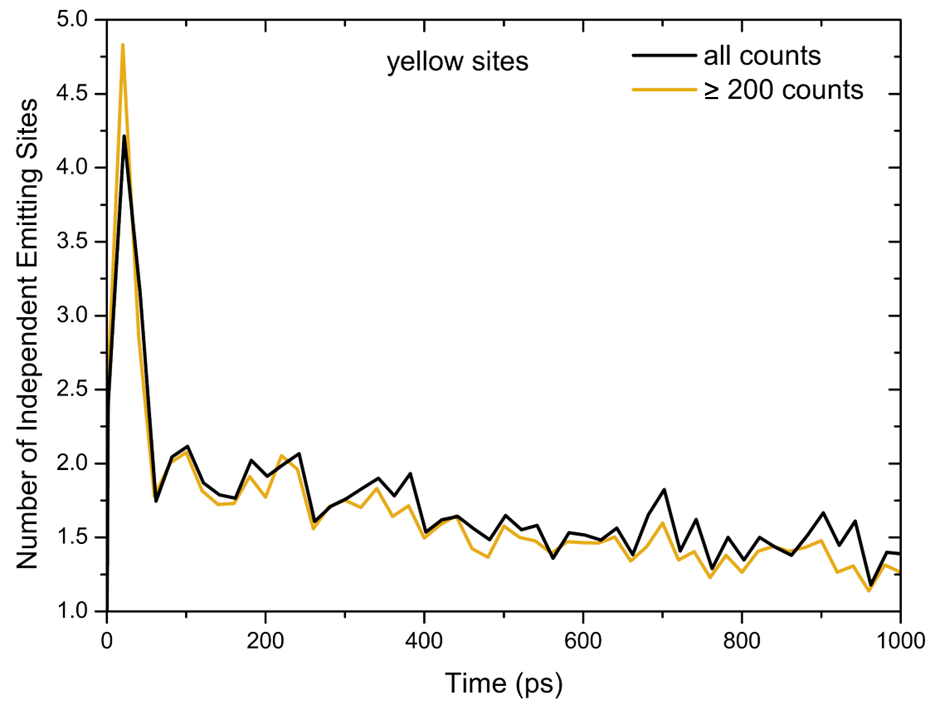


**Supplementary Figure 20:** TRAB plot for yellow sites (colour ratio 0.2 to 0.7) for two count conditions (all photons per 20 ms and ≥ 200 photons per 20 ms).


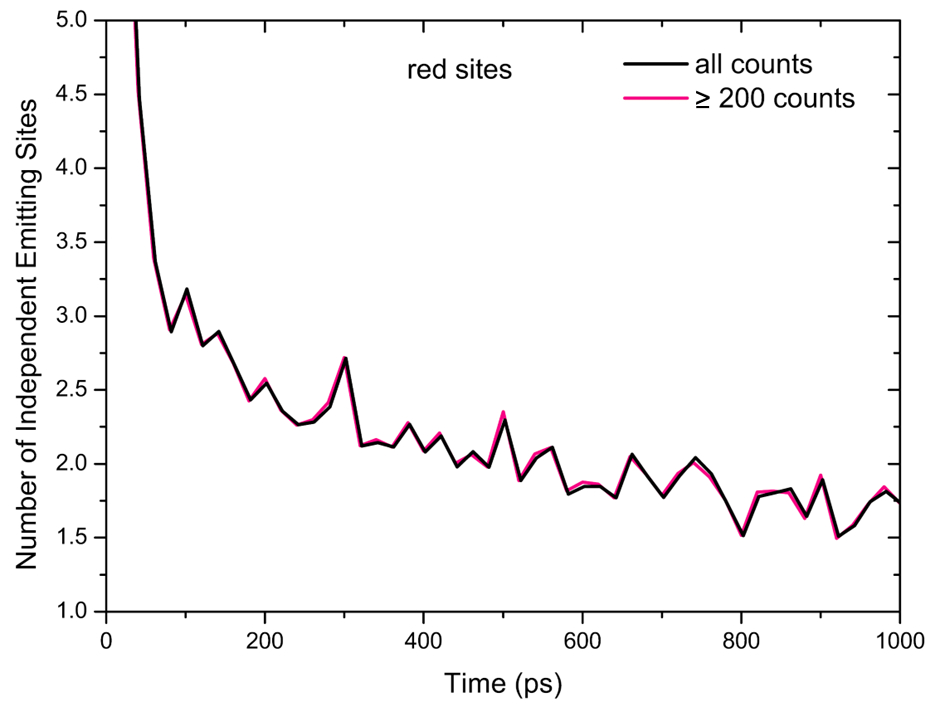


**Supplementary Figure 21:** TRAB plot for red sites (colour ratio 0.8 to 1) for two count conditions (all photons per 20 ms and ≥ 200 photons per 20 ms).

**Supplementary Note 13: GPC of poly(9,9’-dioctylfluorene)**

Gel permeation chromatography (GPC) of PFO was conducted in THF at 35 °C using a column system with an Agilent PL Gel Guard Column (5 µm) and an Agilent PL Gel Mixed-D Column (5 µm) as well as an Agilent Infinity1260 II RID and calibration with narrow poly(styrene) standards.

Two peaks are observed, with the dominant one giving M_n_ of 15100 g mol^-1^ and M_w_ of 30400 g mol^-1^, and a dispersity (Ð) of 2.01. A much smaller second peak at higher molecular weight gives M_n_ of 471000 g mol^-1^ and M_w_ of 555000 g mol^-1^ and Ð of 1.18. The distribution across both peaks thus gives M_n_ of 15300 g mol^-1^ and M_w_ of 39300 g mol^-1^, and Ð of 2.56. The measured elugram and molecular weight distribution are shown below in Supplementary Figures 22 and 23.





**Supplementary Figure 22:** GPC elugram for PFO sample measured in THF at 35 °C.





**Supplementary Figure 23:** PFO molecular weight distribution calculated from GPC measurement in THF at 35 °C according to poly(styrene) calibration, showing dominant low weight fraction and small higher weight one, as described above.
