# Supplementary figures and images for: Highly efficient exciton-exciton annihilation in single conjugated polymer chains
Source: Nat Commun. 2026 Jan 6;17:731. doi: 10.1038/s41467-025-67422-z (PMC12820388; doi:10.1038/s41467-025-67422-z)

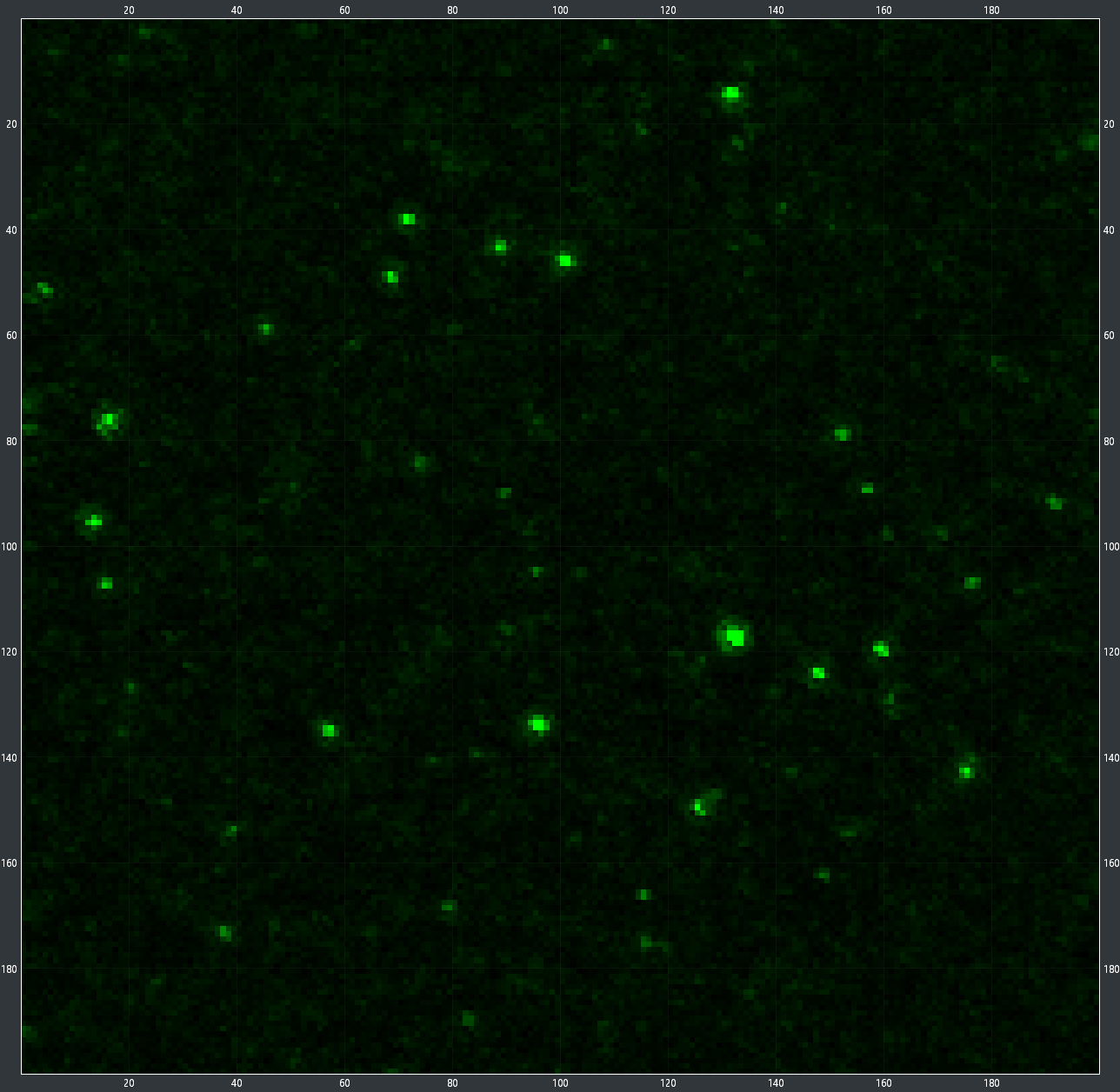

Supplement: Supplementary file 3 — Source data [file 41467_2025_67422_MOESM3_ESM.zip › Main Figs/Fig1/Fig1b/SMS Image - 21Oct2022Im11.png]

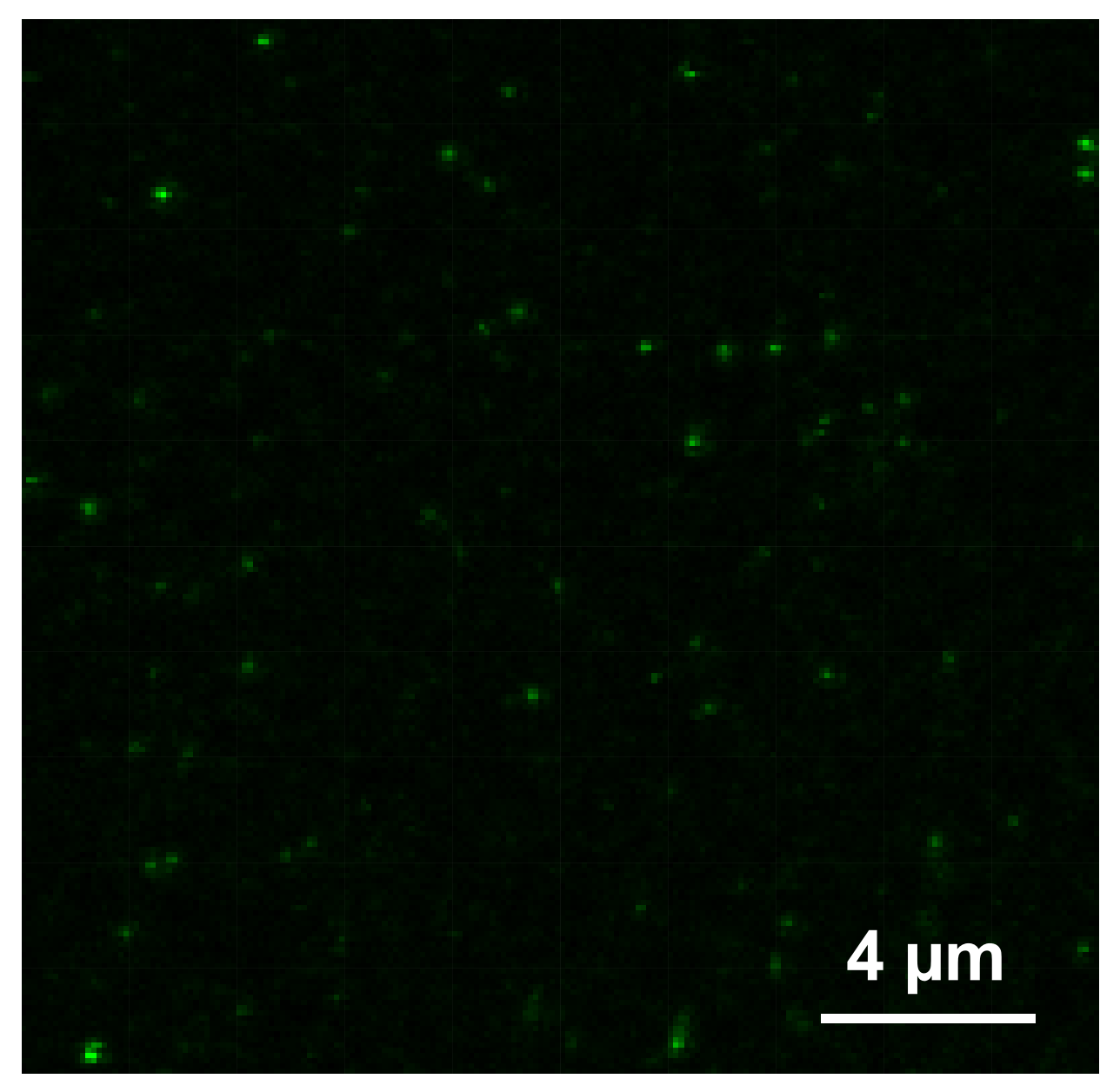

Supplement: Supplementary file 3 — Source data [file 41467_2025_67422_MOESM3_ESM.zip › SI/FigS1/SMS Image - 23062023Im21/23062023-Im21.png]
